# The Association Between COVID-19 Vaccination Uptake and Information-Seeking Behaviors Using the Internet: Nationwide Cross-Sectional Study

**DOI:** 10.2196/59352

**Published:** 2025-01-14

**Authors:** Kazuya Taira, Misa Shiomi, Takayo Nakabe, Yuichi Imanaka

**Affiliations:** 1 Human Health Sciences Graduate School of Medicine Kyoto University Kyoto Japan; 2 Department of Healthcare Economics and Quality Management School of Public Health, Graduate School of Medicine Kyoto University Kyoto Japan; 3 The Database Center of the National University Hospitals University of Tokyo Hospital Tokyo Japan; 4 Department of Health Security System Centre for Health Security, Graduate School of Medicine Kyoto University Kyoto Japan

**Keywords:** COVID-19 vaccines, internet use, information seeking behavior, Japan, vaccine, COVID-19, behavior, panel study, longitudinal, survey, regression analysis, chi-square test, adult, epidemiology, health informatics

## Abstract

**Background:**

The COVID-19 pandemic, declared in March 2020, profoundly affected global health, societal, and economic frameworks. Vaccination became a crucial tactic in combating the virus. Simultaneously, the pandemic likely underscored the internet’s role as a vital resource for seeking health information. The proliferation of misinformation on social media was observed, potentially influencing vaccination decisions and timing.

**Objective:**

This study aimed to explore the relationship between COVID-19 vaccination rates, including the timing of vaccination, and reliance on internet-based information sources in Japan.

**Methods:**

Using a cross-sectional study design using a subset of panel data, this nationwide survey was conducted in 7 waves. A total of 10,000 participants were randomly selected through an internet survey firm, narrowing down to 8724 after applying inclusion and exclusion criteria. The primary outcome was the COVID-19 vaccination date, divided into vaccinated versus unvaccinated and early versus late vaccination groups. The main exposure variable was the use of internet-based information sources. Control variables included gender, family structure, education level, employment status, household income, eligibility for priority COVID-19 vaccination due to pre-existing medical conditions, and a health literacy scale score. Two regression analyses using generalized estimating equations accounted for prefecture-specific correlations, focusing on vaccination status and timing. In addition, chi-square tests assessed the relationship between each information source and vaccination rates.

**Results:**

Representing a cross-section of the Japanese population, the regression analysis found a significant association between internet information seeking and higher vaccination rates (adjusted odds ratio [aOR] 1.42 for those younger than 65 years; aOR 1.66 for those aged 65 years and older). However, no significant link was found regarding vaccination timing. Chi-square tests showed positive associations with vaccination for television, government web pages, and web news, whereas blogs and some social networking sites were negatively correlated.

**Conclusions:**

Internet-based information seeking is positively linked to COVID-19 vaccination rates in Japan, underscoring the significant influence of online information on public health decisions. Nonetheless, certain online information sources, including blogs and some social networks, negatively affected vaccination rates, warranting caution in their use and recognition. The study highlights the critical role of credible online sources in public health communication and the challenge of combating misinformation on less regulated platforms. This research sheds light on how the digital information landscape influences health behaviors, stressing the importance of accurate and trustworthy health information amidst global health emergencies.

## Introduction

### COVID-19 Pandemic and Vaccination

The COVID-19 pandemic, declared by the World Health Organization in March 2020 [1], has had an unprecedented impact globally, affecting billions of lives and causing widespread health, social, and economic disruptions. Vaccination against COVID-19 emerged as a pivotal strategy in controlling the virus’s spread, with over 10 billion vaccine doses administered worldwide by the end of 2022 [2]. The pandemic’s toll has been significant, infecting more than 30 million people and resulting in over 70,000 deaths [3]. In addition, 104,736,436 people, or 80.7% of the population, had received their first vaccine dose by this study [4].

### The Role of the Internet in Public Health and Theoretical Framework

During the COVID-19 pandemic, the way individuals sought health information underwent significant changes. The internet became an indispensable tool, playing a crucial role in public health by serving as the primary means by which people could register for vaccination [5,6]. This shift in information-seeking behavior is essential for understanding the public’s response to health crises [7,8]. Meanwhile, the World Health Organization introduced a framework to address infodemics, characterized by the widespread dissemination of inaccurate information through digital and physical channels [9]. Similarly, the National Academy of Medicine offered recommendations to combat misinformation and disinformation on social media [10]. Indeed, the internet’s influence on health behavior is profound, with misinformation notably undermining confidence in COVID-19 vaccinations [11].

This study is based on the Digital Divide Theory [12] and the Health Belief Model [13]. Digital Divide Theory indicates that disparities in Internet access and digital literacy are related to differences in access to health information and health behaviors. This theory encompasses not only the differences in physical access but also the concept of the second-level divide, which includes the necessary skills and literacy of users [14]. In addition, the Health Belief Model emphasizes factors that influence individuals to engage in health behaviors, such as perceived risk, perceived benefits, perceived barriers, and cues to action. Our research is based on the idea that individuals who can gather information through the internet are more likely to understand the risks and benefits of vaccination and, as a result, experience behavior change.

### Information-Seeking Behavior Using the Internet and Vaccination

The association between vaccination uptake and information-seeking behaviors using the internet is a critical area of study, particularly in the context of the COVID-19 pandemic. Previous research has demonstrated the significant impact of online health information on vaccination behaviors across different vaccines [15-20]. For instance, individuals who actively seek formal health information from credible online sources and engage with health care providers are more likely to get vaccinated in the influenza vaccine [15]. Intervention studies have been conducted on Tetanus, diphtheria, acellular pertussis, and influenza vaccination during pregnancy, and it has also been reported that web-based vaccination information, including websites and interactive social networking services (SNS), can improve vaccination uptake [16]. Conversely, reliance on SNS for vaccination information has been associated with lower vaccination rates, such as the pertussis vaccine uptake during pregnancy, indicating that informal online sources may contribute to vaccine hesitancy [17]. Other studies have also yielded mixed results, with internet information gathering having a positive effect on pneumococcal vaccination of adults with heart diseases [18] and a negative effect on a mother’s decision to vaccinate her daughter with human papillomavirus [19,20]. These findings underscore the complexity of online information’s role in vaccination behavior and highlight the necessity of distinguishing between formal and informal sources to understand their differing impacts fully.

For COVID-19 vaccination, a study indicates that the behavior of seeking health information on the internet is positively linked to vaccination uptake in China [21]. Various studies have identified vaccination hesitancy as a concern, noting a reduction in hesitancy when the internet is the primary source of information about COVID-19 [22]. Conversely, some studies have highlighted the negative effects of social media on vaccine hesitancy [23,24]. In Japan, reliance on internet news and video sharing sites was found to exacerbate hesitancy toward COVID-19 vaccination among individuals younger than 65 years [25].

These findings indicate that the effects of internet-based information sources on COVID-19 vaccination uptake are mixed and inconclusive. There is a need for further analysis, as the impact appears to vary depending on the type of internet information sources, such as web news, social media, and video sharing sites. In addition, no studies have reported an association with the timing of vaccination, whether late or early, or with the presence or absence of vaccination.

This study aims to investigate the association between COVID-19 vaccination uptake or timing and the use of internet-based information sources in more detail.

## Methods

### Study Design and Participants’ Setting

This study used a cross-sectional study design using a subset of panel data, conducting nationwide web-based surveys 7 times from the first wave (October to November 2020) to the seventh wave (February to March 2022). The surveys were closed surveys facilitated through an internet research company, Rakuten Insight Inc, using their proprietary web platform and a self-administered, anonymized web form. The data across these panels were linked using user identification numbers known only to the research company. Rakuten Insight is among the largest internet research services, boasting 2.2 million registered active users as of September 2022. In this study, only complete cases with no unanswered items were collected, and multiple responses from the same person were eliminated using panel monitor registration information and IP addresses. Primarily, this study used data from the sixth panel (survey conducted between November 5 and 25, 2021), which coincided with the conclusion of the public’s initial COVID-19 vaccination phase.

The survey initially sampled 10,000 participants, ensuring an equal distribution across gender, age, and prefectures through random selection during the first wave. If participants dropped out in subsequent surveys after the second wave, resampling targeted individuals who had responded in any of the previous waves. Should a shortfall persist, new samples were recruited, with replacements selected from the closest matching age group within the same prefecture and gender category. In the event of dropouts during the follow-up survey, new samples were added as necessary to maintain a total of 10,000 participants for each panel survey. The decision to establish a sample size of 10,000 was informed by the need for random sampling to ensure equitable allocation and representativeness, taking into account the distribution and prevalence of monitors. This sample size also facilitates effective stratification across age, gender, and prefectural demographics, enabling robust multivariate analysis.

### Inclusion and Exclusion Criteria

The eligibility criteria for the survey, aimed at examining the psychological landscape of ethical values and anxiety, required participants to be adults aged 20 years or older. This age threshold was established to ensure the reliability of the feedback received. Furthermore, to reduce sampling bias inherent in internet-based surveys, professionals working in survey or advertising agencies were excluded from participation, which was in line with the regulations set by internet survey companies. The variable related to medical history was cross-referenced using user IDs from the fifth survey panel, conducted between September and October 2021. As a result, any cases where this variable was missing were systematically excluded from the analysis. Medical and caregiving professionals were also excluded from the analysis because they received vaccinations before the general public and possessed specialized expertise and unique access to information. In addition, participants who reported their vaccination dates as being before April 14, 2021 (the start date of mass vaccination) were excluded, as these entries were considered input errors.

### Measures

#### Main Outcome

The primary outcome variable was the date of COVID-19 vaccination. This variable was converted into two binary variables: (1) one indicating whether individuals were vaccinated or not (vaccination uptake) and (2) the other differentiating between dates before and after the 75th percentile (vaccination timing).

In this study, the dependent variable of vaccination timing is defined based on Everett Rogers’ Diffusion of Innovation theory [26]. According to this theory, the adoption of new innovations follows a specific pattern, and the population is categorized into 5 adopter categories that consist of innovators, early adopters, early majority, late majority, and laggards.

Given the unprecedented speed and scale of the COVID-19 vaccination rollout, we aimed to identify the segment of the population that adopted the vaccination last, corresponding to the “Laggards” category in Rogers’ theory. This category is generally characterized by lower socioeconomic status, personality challenges, and limited social interaction, making its identification meaningful. Statistical methods such as mean and SD are proposed to determine the Laggards category. Considering the timing that most elderly individuals had completed their vaccinations and younger individuals were starting theirs, we determined that defining this group using the fourth quartile was more appropriate.

#### Exposure

The exposure variable was identified as the source of information related to COVID-19. Participants were asked to select their sources of information from a list provided in a multiple-choice format (Textbox 1). Based on their responses, the options were categorized into two groups, those that involved information seeking through the internet and those that did not. The “with Internet” category included participants who selected at least one of the following options, Twitter, Facebook (Meta), LINE News (LY Corporation), other social networking services, YouTube (Google), COCOA (COVID-19 Contact-Confirming Application, Ministry of Health, Labour and Welfare), web pages of local governments or the Ministry of Health, Labour and Welfare, web pages of professional associations, blogs by medical professionals, personal blogs (nonprofessional), and web news. Participants who selected only options outside of these were classified as engaging in information seeking without the internet.

**Ways of obtaining COVID-19–related information.**

Television, Radio, Magazines/Books, Asahi Shimbun, Mainichi Shimbun, Yomiuri Shimbun, Sankei Shimbun, Nikkei Shimbun, Other newspapers, Twitter, Facebook, LINE news, Other social networking services, YouTube, COCOA (COCOA is an abbreviation for COVID-19 Contact-confirming Application, a smartphone application produced by the Japanese government for COVID-19 measures.), WEB pages of local governments or Ministry of Health, Labour and Welfare, WEB pages of professional organizations, Blogs of medical professional, Blogs of personal (nonprofessional), WEB news, Information from friends and acquaintances, Information from family members, Others

#### Control Variables

The control variables included gender, family structure, educational background, employment status, household income, pre-existing medical conditions eligible for priority COVID-19 vaccination (Multimedia Appendix 1), and the 14-item health literacy scale for Japanese adults (HLS-14) [27]. Family structure was categorized as either living alone or living with family members. Educational background was classified into three categories, less than a bachelor’s degree, associate or bachelor’s degree or higher, and unwilling to answer. Household income was defined as no income, less than 3 million yen (approximately US $19,355, at an exchange rate of 155 yen/US dollar), 3 million yen to 5 million yen (approximately US $19,355 to $32,258), 5 million yen to 8 million yen (approximately US $32,258 to $51,613), 8 million yen (approximately US $51,613) or more, or unwilling to answer. The HLS-14 measures health literacy across 3 domains: functional health literacy, communicative health literacy, and critical health literacy. Respondents rate each item on a 1 to 5 scale, with total scores ranging from 14 to 70 points; higher scores indicate greater health literacy.

### Statistical Analysis

In Japan, priority for vaccination was given to individuals aged 65 years and older. Therefore, the analysis differentiated between those aged 65 years and older and those younger than 65 years. Descriptive statistics were used to profile the participants and their vaccination statuses. Two regression analyses generalized estimating equations accounted for prefecture-specific correlations, focusing on vaccination uptake and vaccination timing. In these analyses, the main exposure variable was whether information seeking was conducted with or without the internet. The models were adjusted for gender, family structure, educational background, employment status, household income, eligibility for priority COVID-19 vaccination due to pre-existing medical conditions, and HLS-14 scores. In addition, chi-square tests assessed the relationship between the use of each information source and vaccination uptake.

### Ethical Considerations

This study was conducted with the approval of the Kyoto University Medical Ethics Review Committee (approval number: R2670). Informed consent was obtained through the initial screen of the online survey, where participants were informed that completion of the questionnaire implied consent. The data provided to the researchers by the commissioned internet research company was anonymized, ensuring no personally identifiable information was included. Participants received compensation in the form of points from the survey company, in accordance with their internal regulations. The exact number of points awarded was not disclosed to the researchers, as this information is confidential.

## Results

### Participants Included in the Analysis

**Figure 1:**
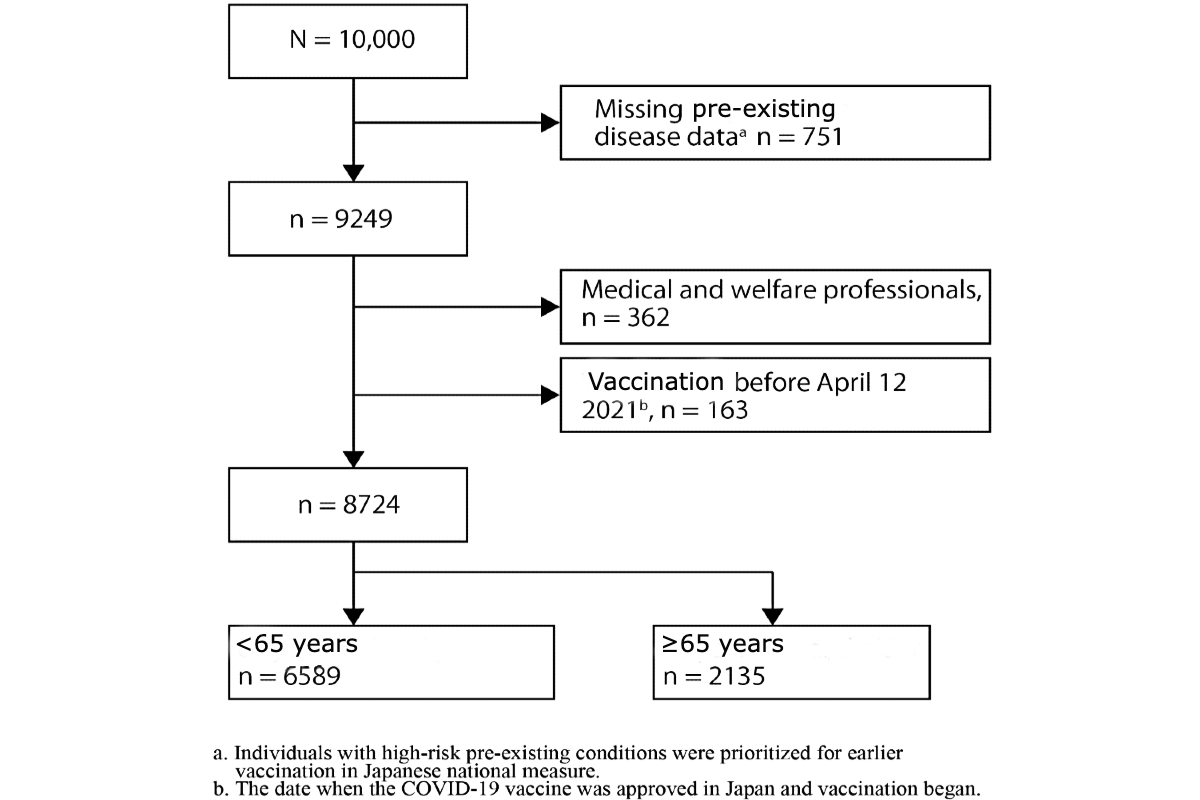
Flowchart of inclusion and exclusion criteria.

The sixth survey, which involved 10,000 respondents, was linked with the data from the fifth survey, which led to the exclusion of 751 cases due to missing information on pre-existing medical conditions. In addition, further exclusions were made, 362 individuals working in medical or caregiving professions and 163 participants who reported vaccination dates before April 12, 2021, were deemed ineligible. As a result, the final sample size for analysis was 8724 participants, comprising 6589 individuals younger than 65 years of age and 2135 aged 65 years and older (Figure 1).

### Demographic Characteristics of the Participants

The analysis of demographic characteristics among the participants showed that of the 6589 individuals younger than 65 years, 3260 (49.5%) were women, with a median age of 44 (IQR 33-55). Among the 2135 respondents aged 65 years and older, 1009 (47.3%) were women, with a median age of 71 (IQR 68-73; Table 1). The use of internet-based information sources did not significantly vary between respondents younger than 65 years and those aged 65 years and older. A total of 42.0% (2765/6589) of participants younger than 65 years and 37.0% (791/2135) of those aged 65 and older used 2 or more sources of information. Television was the most popular information source, used by 86.3% (5684/6589) of respondents younger than 65 years and 94.0% (2006/2135) of the older group. Although there was no significant difference in the types of internet sources used, younger participants were more inclined to use social networking services such as Twitter and LINE news (Multimedia Appendix 2).

**Table 1:** Participants’ demographic characteristics.

			Younger than 65 years							65 years or older				
			Total, (n=6589)		Vaccinated, (n=5325)		Not vaccinated, (n=1264)		Total, (n=2135)			Vaccinated, (n=1921)		Not vaccinated, (n=214)
Female gender, n (%)			3260 (49.5)		2644 (49.7)		616 (48.7)		1009 (47.3)			885 (46.1)		124 (57.9)
Age (years), median (IQR)			44 (33-55)		45 (34-55)		40.5 (29-50)		71 (68-73)			71 (68-73)		71 (68-73)
Health literacy^a^, median (IQR)			47 (42-52)		47 (42-53)		43 (42-50)		47 (42-52)			48 (43-53)		45 (42-50.8)
**Family structure, n (%)**														
	With family members	5414 (82.2)		4427 (83.1)		987 (78.1)		1787 (83.7)			1624 (84.5)		163 (76.2)	
	Living alone	1175 (17.8)		898 (16.9)		277 (21.9)		348 (16.3)			297 (15.5)		51 (23.8)	
**Educational background, n (%)**														
	Less than a bachelor’s degree	3138 (47.6)		2479 (46.6)		659 (52.1)		1173 (54.9)			1039 (54.1)		134 (62.6)	
	Associate or bachelor’s degree or higher	3205 (48.6)		2679 (50.3)		526 (41.6)		922 (43.2)			848 (44.1)		74 (34.6)	
	Unanswered	246 (3.7)		167 (3.1)		79 (6.3)		40 (1.9)			34 (1.8)		6 (2.8)	
**Household income, n (%)**														
	No income	158 (2.4)		109 (2.0)		49 (3.9)		23 (1.1)			19 (1.0)		4 (1.9)	
	<3 million yen (approximately US $19,355)	1211 (18.4)		943 (17.7)		268 (21.2)		633 (29.6)			547 (28.5)		86 (40.2)	
	3-4 million yen (approximately US $19,355 to 32,258)	1426 (21.6)		1113 (20.9)		313 (24.8)		636 (29.8)			580 (30.2)		56 (26.2)	
	5-7 million yen (approximately US $32,258 to 51,613)	1554 (23.6)		1312 (24.6)		242 (19.1)		285 (13.3)			262 (13.6)		23 (10.7)	
	≥8 million yen (approximately US $51,613)	1068 (16.2)		945 (17.7)		123 (9.7)		154 (7.2)			145 (7.5)		9 (4.2)	
	Unanswered	1172 (17.8)		903 (17.0)		269 (21.3)		404 (18.9)			368 (19.2)		36 (16.8)	
**Risk of medical history for severe COVID-19, n (%)**														
	Not high risk	5283 (80.2)		4240 (79.6)		1043 (82.5)		1065 (49.9)			934 (48.6)		131 (61.2)	
	High risk	1306 (19.8)		1085 (20.4)		221 (17.5)		1070 (50.1)			987 (51.4)		83 (38.8)	
**Employment status, n (%)**														
	Not employed	1130 (17.1)		883 (16.6)		247 (19.5)		954 (44.7)			844 (43.9)		110 (51.4)	
	Employed	5459 (82.9)		4442 (83.4)		1017 (80.5)		1181 (55.3)			1077 (56.1)		104 (48.6)	
**Internet use for information seeking behavior, n (%)**														
	Does not use	1957 (29.7)		1477 (27.7)		480 (38.0)		700 (32.8)			602 (31.3)		98 (45.8)	
	Does use	4632 (70.3)		3848 (72.3)		784 (62.0)		1435 (67.2)			1319 (68.7)		116 (54.2)	

^a^HLS-14: 14-item health literacy scale.

### Summary of Vaccination Uptake as Outcome Variables

**Figure 2:**
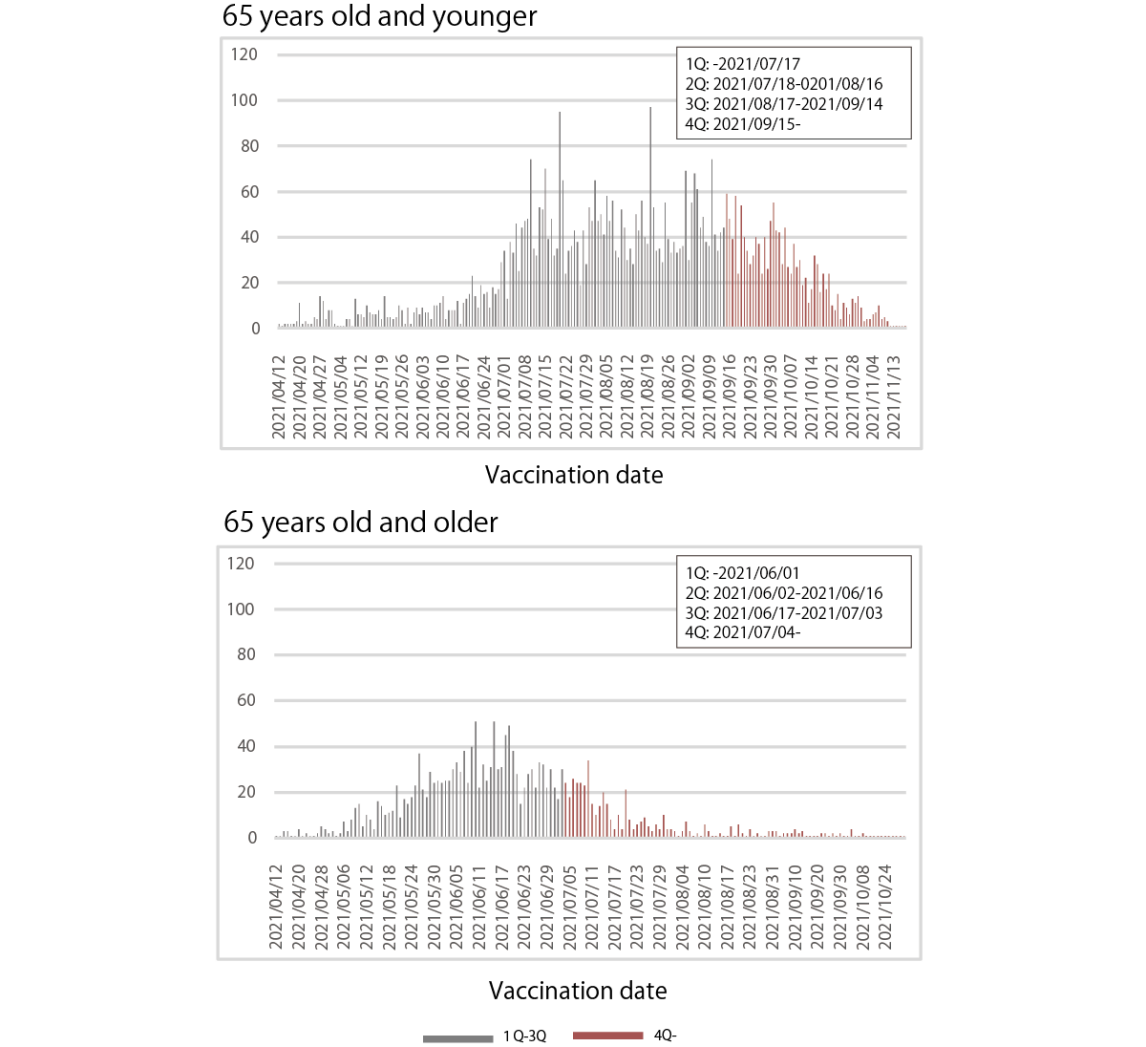
Bar chart showing vaccination timing.

Regarding vaccination uptake, 5325 individuals (80.8%, 5325/6589) younger than 65 years and 1921 (90.0%, 1921/2135) aged 65 years and older had been vaccinated. Internet-based information gathering was undertaken by 4632 individuals (70.3%, 4632/5689) younger than 65 years and 1435 (67.2%, 1435/2135) aged 65 or older (Table 1). Furthermore, the quartile values for vaccination dates for those younger than 65 years were: 25th percentile on July 7, 2021, 50th percentile on August 17, 2021, and 75th percentile on September 15, 2021; for those aged 65 years and older, the values were 25th percentile on June 2, 2021, 50th percentile on June 17, 2021, and 75th percentile on July 4, 2021. Consequently, regarding the vaccination timing variable, participants were classified as part of the delayed vaccination group if their vaccination occurred after September 15, 2021, for those younger than 65 and after July 4, 2021, for those aged 65 years and older (Figure 2).

### Regression Analysis Results

In the regression analysis with vaccination uptake as the outcome, seeking information through the internet was significantly associated with higher vaccination rates compared with not using the internet for information seeking (adjusted odds ratio [aOR] 1.42, 95% CI 1.27-1.60 for those younger than 65 years; aOR 1.66, 95% CI 1.16-2.37 for those aged 65 years and older). However, when examining vaccination timing as the outcome, there was no significant relationship between using the internet for information and delayed vaccination (aOR 1.12, 95% CI 0.96-1.31 for those younger than 65 years; aOR 1.25, 95% CI 0.98-1.59 for those aged 65 years and older; Table 2).

**Figure 3:**
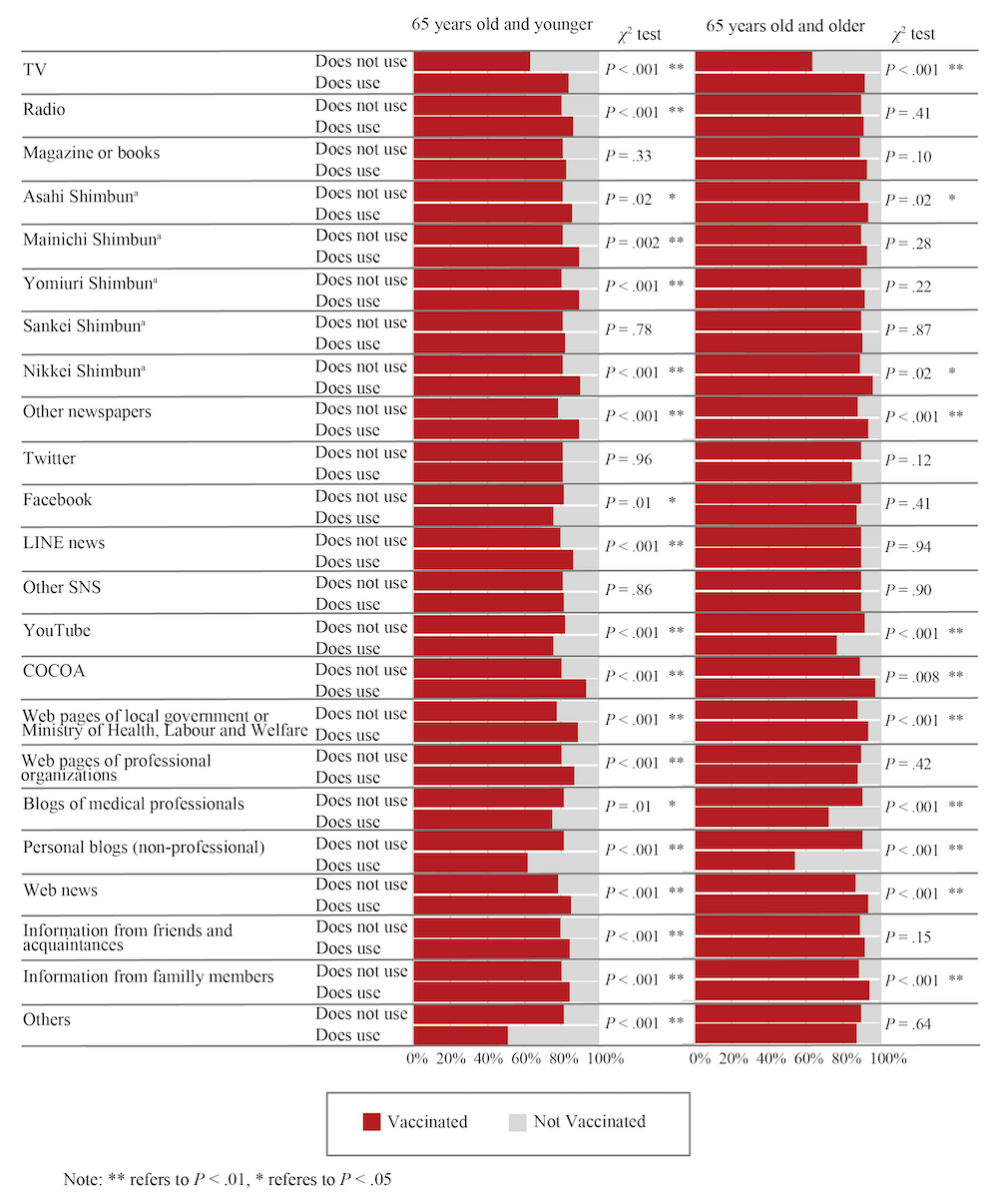
Results of chi-square tests between the information resource and vaccination uptake. “Shimbun” refers to a Japanese newspaper. COCOA: COVID-19 Contact-confirming Application; SNS: social networking services.

Chi-square tests assessing the relationship between the use of each information resource and vaccination uptake revealed that TV, government and professional web pages, and web news were more positively associated with COVID-19 vaccination. Conversely, blogs, YouTube, and some social networking sites had a negative association with vaccination uptake (Figure 3).

**Table 2:** Results of regression analysis using generalized estimating equation model.

Outcomes		Vaccination uptake（Yes=1, No=0)		Vaccination timing（1Q-3Q=1, 4Q=0)	
		Younger than 65 years, aOR^a^ (95% CI)	65 years old and older, aOR (95% CI)	Younger than 65 years, aOR (95% CI)	65 years old and older, aOR (95% CI)
**Gender, median (IQR)**					
	Women	Reference	Reference	Reference	Reference
	Men	1.19 (1.05-1.34)^b^	0.80 (0.53-1.19)	1.01 (0.90-1.14)	0.99 (0.78-1.24)
Health literacy (HLS-14)^c^		1.03 (1.02-1.03)^b^	1.02 (1.00-1.05)^b^	1.01 (1.00-1.02)^b^	1.01 (1.00-1.03)
**Family structure**					
	With family member	Reference	Reference	Reference	Reference
	Living alone	0.8 (0.68-0.94)	0.69 (0.47-1.01)	0.89 (0.74-1.06)	1.04 (0.75-1.45)
**Educational background**					
	Less than a bachelor’s degree	Reference	Reference	Reference	Reference
	Associate or bachelor’s degree or higher	0.83 (0.72-0.96)	0.89 (0.64-1.25)	0.78 (0.69-0.88)^b^	0.85 (0.69-1.06)
	Unanswered	1.47 (1.11-1.97)^b^	1.50 (0.62-3.65)	1.35 (0.97-1.88)	1.03 (0.52-2.03)
**Household income**					
	No income	Reference	Reference	Reference	Reference
	<3 million yen (approximately US $19,355)	0.76 (0.53-1.08)	0.87 (0.30-2.52)	0.71 (0.43-1.17)	0.62 (0.24-1.59)
	3-4 million yen (approximately US $19,355 to 32,258)	0.78 (0.53-1.15)	0.67 (0.23-1.96)	0.82 (0.52-1.3)	0.53 (0.19-1.49)
	5-7 million yen (approximately US $32,258 to 51,613)	0.74 (0.5-1.08)	0.63 (0.18-2.23)	0.72 (0.46-1.13)	0.66 (0.23-1.90)
	≥8 million yen (approximately US $51,613)	0.41 (0.26-0.64)^b^	0.77 (0.12-4.97)	0.55 (0.34-0.88)^b^	0.56 (0.20-1.61)
	Unanswered	0.75 (0.51-1.10)	0.56 (0.19-1.66)	0.66 (0.45-0.97)^b^	0.54 (0.20-1.44)
**Risk of medical history for severe COVID-19**					
	Not high risk	Reference	Reference	Reference	Reference
	High risk	1.21 (1.03-1.42)^b^	1.49 (1.17-1.88)^b^	1.78 (1.51-2.11)^b^	1.17 (0.93-1.46)
**Employment status**					
	Not employed	Reference	Reference	Reference	Reference
	Employed	0.82 (0.67-1.01)	0.93 (0.66-1.29)	1.20 (1.02-1.42)^b^	1.28 (1.03-1.58)^b^
**Internet use for information seeking behavior**					
	Does not use	Reference	Reference	Reference	Reference
	Does use	1.42 (1.27-1.60)^b^	1.66 (1.16-2.37)^b^	1.12 (0.96-1.31)	1.25 (0.98-1.59)

^a^aOR: adjusted odds ratio.

^b^Items with a 95% CI not crossing 1 are considered statistically significant (*P*<.05).

^c^HLS-14: The 14-item health literacy scale.

## Discussion

### Principal Results

This study offers a thorough analysis of the relationship between COVID-19 vaccination uptake and the use of various online information resources. Our results indicate that seeking information on the internet is positively associated with higher vaccination rates, highlighting the significant influence of online information on public health decisions. Interestingly, however, our data did not reveal a significant link between online information seeking and the timing of vaccination, suggesting that while online resources contribute to the decision to get vaccinated, they may not affect how promptly individuals act on this decision.

The further analysis presented in Figure 3 showcases the varied impact of different internet resources. Government web pages, for instance, positively influenced vaccination decisions, emphasizing the critical role of credible, official sources in public health communication. On the contrary, information from blogs and SNS was found to have a negative effect. This highlights concerns about the spread of misinformation on less regulated platforms and underlines the necessity for more efficient measures to counteract misleading health information online. Considering the usage rates of each media type, TV emerged as a predominant information source, with 86.3% (5684/6589) of participants younger than 65 years and 94.0% (2006/2135) of those 65 years and older. This widespread use of TV suggests that even excluding TV’s influence, internet usage would likely still be statistically significant in relation to vaccination uptake. Nevertheless, the potential interactive effects between television and specific online sources call for more detailed future investigations.

The insights gained from this study enhance our understanding of how the digital information landscape affects health behaviors and underscore the urgent need to ensure access to accurate and trustworthy health information during global health emergencies.

### Comparison With Previous Work

The findings of this study align with those of previous research, indicating that obtaining COVID-19 information on the internet is positively correlated with vaccination uptake [21,28]. Similarly, concerning vaccination hesitancy, the results consistently showed that internet information-seeking behavior plays a positive role in reducing hesitancy [22,23]. While it is plausible that the type of information disseminated across various media, including the internet, may vary by country due to cultural and linguistic differences, these results suggest that the trends observed in Japan mirror those in other nations. However, a study by Hori et al [25] in Japan found that internet web news negatively affected vaccination hesitancy, diverging from some findings of this study. Despite using the same internet research firm for sampling, the exclusion criteria between the two studies significantly differed. Notably, focusing on specific target populations—excluding individuals aged 65 years and older, those who are obese, have pre-existing medical conditions, or have a clear intention regarding vaccination—might yield different impacts on COVID-19 vaccination outcomes. Many of these studies have been conducted in developed economies such as the United States, China, South Korea, Japan, and European Union countries [21-23,25,28], and different results could be obtained if studies were conducted in more diverse countries, including developing economies. Indeed, a paper tracking changes in search trends during the pandemic on Google Trends also reported that search trends were influenced by central government actions and news sources, with some search terms trending differently in different countries [29].

Moreover, reports on information-seeking behavior during the COVID-19 pandemic have indicated that traditional media such as television are more trusted than social media, and Information-seeking behavior through the internet is associated with higher levels of knowledge [30,31]. With regard to SNSs, there is a risk that the sharing of incorrect information may be accelerated because people who are more likely to share information are more confident in their own information and judgment due to a high degree of information sufficiency and trust in the source of information on SNSs [30]. In fact, the outcomes related to social media usage are consistent with previous findings, indicating a negative effect on vaccination rates [23,32]. The growing number of studies highlighting misinformation on social media [33] and the widespread misinformation on critical public health issues [34] advocate for cautious use of social media to prevent reliance on potentially misleading information [32]. However, it is also true that nearly half of people seek COVID-19 information from SNSs [35,36], and it has been shown that vaccination coverage is higher among those who follow reliable information sources on SNSs, so it is important to have a good relationship with SNSs [35]. Moreover, negative impacts have also been reported concerning video sharing sites [25], with some platforms, like YouTube, implementing measures to curb the spread of inaccurate health information by introducing eHealth services [37]. As recommended by the WHO framework [9], a multifaceted approach involving user literacy and ethics, the provision and translation of information by evidence-based professionals, and the analysis and improvement of services by platform operators is essential for fostering a well-informed society in terms of health information.

Therefore, using the internet can be effective in scenarios where vaccination is encouraged as part of a national strategy, such as the COVID-19 response. However, it is crucial to select reliable sources of information and enhance services to ensure the dissemination of accurate information.

### Strength and Limitations

The strengths of this study include its close alignment with the demographic distribution of the Japanese population, featuring an aging rate of 24.5% (compared with the national average of 28.9%), a women representation of 49.5% (national average: 51.4%) [38], and a vaccination rate of 83.1% (national average: 75.2% as of November 30, 2021 [6]), ensuring representativeness. A unique aspect of this study is its focus on actual COVID-19 vaccination uptake rather than intentions or hesitancy, analyzing the association with various internet information sources in detail. However, the study is not without limitations. First, it did not thoroughly examine the effect of using multiple information sources on vaccination uptake. Second, the context of the COVID-19 vaccination campaign, being a specific situation with national strategies, including a large-scale vaccination effort, requires caution in generalizing these findings to vaccinations for other infectious diseases. Third, the simultaneous administration of vaccines over a brief period reduced the variance in vaccination timing, potentially making it challenging to identify a relationship between vaccination timing and information sources. While the impact of information sources may extend beyond the context of emerging infectious disease pandemics, as similar outcomes have been reported for other infectious diseases like influenza [39], more comprehensive studies are necessary in the future. Finally, since this survey was conducted through an internet web-form, people who do not use the internet at all were excluded from the survey. As a result, the participants may exhibit higher levels of information-gathering behavior through the internet compared with the general population. However, this likely represents a conservative effect on the results of this study, suggesting that the true impact of the exposure may be even stronger. Future research using face-to-face or mail survey methods would be desirable to corroborate these findings. Finally, this study might have potential selection bias due to the nature of an internet survey. Individuals, particularly in 65 years old and older, without access to or interest in internet surveys may differ in vaccination behaviors, and unvaccinated individuals may have been less likely to participate. However, over 16.9% (1478/8724) of respondents reported being unvaccinated, suggesting that arrange of vaccination statuses was adequately represented. Despite this limitation, the impact on the study’s conclusions is likely minimal.

### Conclusions

Seeking information related to COVID-19 on the internet is significantly associated with COVID-19 vaccination uptake but not with the timing of vaccination. However, an analysis differentiated by types of internet media reveals a varied impact across these platforms. Specifically, platforms like blogs and social media networks have been identified as negatively affecting vaccination uptake. Public health policy makers should strategically use online information media during an emerging infectious disease pandemic such as COVID-19, taking into account the unique characteristics of each media type.

## Appendix

Multimedia Appendix 1 Criteria for Prioritizing COVID-19 Vaccination Based on Preexisting Medical Conditions.

Multimedia Appendix 2 Usage status of Information Resources.

## Abbreviations

aOR: adjusted odds ratio
COCOA: COVID-19 Contact-confirming Application
HLS-14: 14-item health literacy scale for Japanese adults
SNS: social networking services

## Data Availability

The data used in this study are not available for redistribution due to a lack of participant consent for sharing with third parties.
