# Supplementary material for: The Association Between COVID-19 Vaccination Uptake and Information-Seeking Behaviors Using the Internet: Nationwide Cross-Sectional Study
Source: J Med Internet Res. 2025 Jan 14;27:e59352. doi: 10.2196/59352 (PMC11775489; doi:10.2196/59352)
Supplement: Multimedia Appendix 1 [file jmir_v27i1e59352_app1.docx]

Appendix 1. Criteria for Prioritizing COVID-19 Vaccination Based on Preexisting Medical Conditions

1: Individuals currently receiving outpatient or inpatient treatment for the following conditions or diseases:

1.1 Chronic Respiratory Diseases

1.2 Chronic Cardiovascular Diseases, including Hypertension

1.3 Chronic Renal Diseases

1.4 Chronic Liver Diseases (e.g., Cirrhosis)

1.5 Diabetes Mellitus under treatment with Insulin or Oral Medication,

or coexisting with other comorbidities

1.6 Hematological Disorders (excluding Iron-deficiency Anemia)

1.7 Immunocompromising Conditions, including ongoing treatment for

malignant tumors

1.8 Those receiving immunosuppressive therapies such as steroids

1.9 Neurological or Neuromuscular Disorders associated with

immunodeficiencies

1.10 Physical dysfunction due to neurological or cardiomyopathic

diseases (e.g., respiratory impairments)

1.11 Chromosomal Abnormalities

1.12 Severe Physical and Intellectual Disabilities (presence of both severe

motor and intellectual impairments)

1.13 Sleep Apnea Syndrome

1.14 Severe Mental Disorders (including those who are hospitalized for

psychiatric treatment, possess a Mental Disability Welfare Certificate,

or qualify as 'severe and persistent' under medical assistance for

outpatient psychiatric care) or Intellectual Disabilities (possession of a

Therapeutic Education Certificate)

2: Individuals with Obesity meeting the criterion (BMI of 30 or higher)
