# Supplementary figures and images for: The Association Between COVID-19 Vaccination Uptake and Information-Seeking Behaviors Using the Internet: Nationwide Cross-Sectional Study
Source: J Med Internet Res. 2025 Jan 14;27:e59352. doi: 10.2196/59352 (PMC11775489; doi:10.2196/59352)

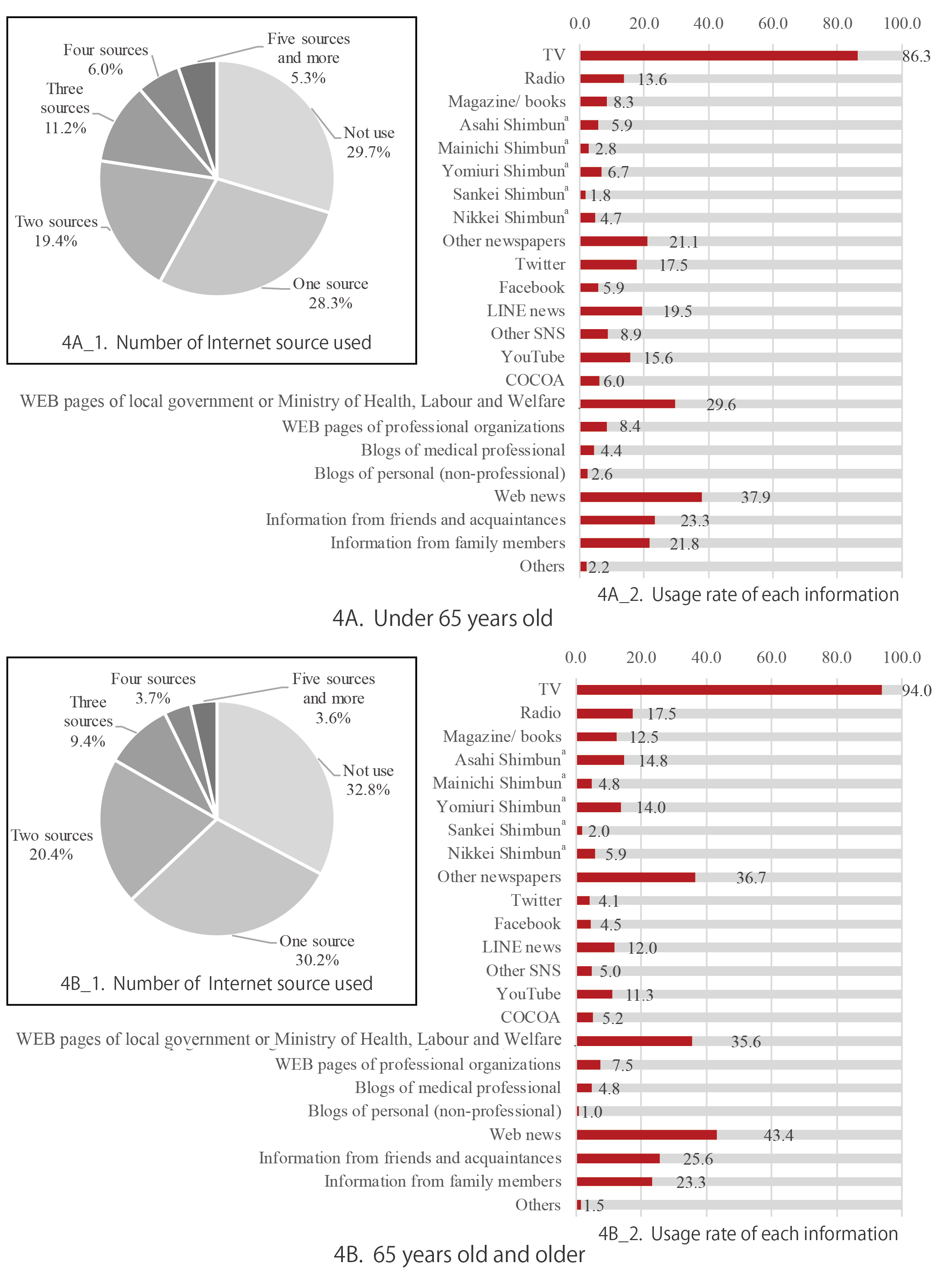


Appendix 2. Usage status of Information Resources

1. "Shimbun" refers to a Japanese newspaper.

Supplement: Multimedia Appendix 2 [file jmir_v27i1e59352_app2.docx]
